# 

**Published:** 1858-06

**Authors:** 


### CONTENTS, No. 4, Vol. IV.

ORIGINAL COMMUNICATIONS.

| | Page. |
|---|---|
| Capillary Circulation, D. L. McGugin, M. D. - | 241 |
| Two Cases of Puerperal Convulsions, J. Williamson, M. D. - - - - - - | 252 |
| Case of Pulmonary Apoplexy, O. W. Newell, M. D. | 257 |

DEPARTMENT OF SELECTIONS.

| | |
|---|---|
| Convulsions, Their Pathology and Treatment. - | 260 |
| What they think of us. - - - - - | 268 |
| Clinical Lecture. - - - - - - - | 272 |
| On the use of Iodide of Potassium in the Treatment of Luchorrhœa by Injections. - - - | 280 |
| American Medical Association. - - - | 282 |
| State Medical Society. - - - - - - | 307 |
| Keokuk County Medical Society, - - - - | 311 |

EDITORIAL DEPARTMENT.

| | |
|---|---|
| State Medical Society. - - . . | 311 |
| Ninth Annual Announcement of Medical Department Iowa University. - - - - | 313 |

